# Currarino’s triad diagnosed in an adult woman

**DOI:** 10.1007/s00586-012-2311-2

**Published:** 2012-04-24

**Authors:** Lotte M. E. Berghauser Pont, Clemens M. F. Dirven, Ruben Dammers

**Affiliations:** Department of Neurosurgery, Erasmus MC, ‘s Gravendijkwal 230, Office Hs-114, P.O. Box B2040, 3000 CA Rotterdam, The Netherlands

**Keywords:** Currarino, Anorectal malformation, Sacrococcygeal agenesis, Presacral mass, Meningocele

## Abstract

**Purpose:**

To report on a female patient diagnosed with Currarino’s triad in adulthood.

**Case report:**

This case presents an adult patient with a medical history of a congenital anal atresia, a partial sacral agenesis, and a surgically treated ectopic anus. After a coincidentally observed presacral mass by MRI, due to unexplained constipation later in adulthood, Currarino’s triad was suspected in this patient. This triad consists of anorectal malformation(s), sacrococcygeal defects and a presacral mass of various origin. Further investigation confirmed the mass to be a meningocele, and showed a tethered cord and a syrinx.

**Conclusions:**

In (young) patients with anorectal malformations, although having no other symptoms, further examination might be required to exclude Currarino’s triad. Importance of early diagnosis and multidisciplinary assessment is recommended to establish adequate treatment if needed.

## Introduction

Constipation is a symptom commonly observed in neonates. Few patients suffering from constipation at young infancy, however, are diagnosed with Currarino’s triad, consisting of a malformation in the anorectal region, a (partial) sacral agenesis, and the occurrence of a presacral mass. It is caused by a deletion on chromosome 7q36 [1]. Since this deletion is rather scarce, it is difficult to obtain an accurate incidence. Isik et al. described 1.7 % patients with Currarino in a cohort of 234 cases with lipomeningomyelocele [2]. Up to date, about 270 cases have been reported since the pediatric radiologist Currarino and co-authors have described this triad in 1981 [3]. Earlier, in 1926 and 1974, similar cases have been described [4]. Although being a rare condition, large multicenter series have been published that intend to give more insight into the genetic aspects of Currarino’s triad. Currarino’s triad has several important diagnostic difficulties. Since the treatment decisions are predominantly based on the radiology, these aspects need to be examined carefully. The clinical, i.e., neurological state also plays a major part in decision making, although in case of threatening but asymptomatic abnormalities on radiological assessment, surgery might nevertheless be proposed.

In this report, we present an adult female with a history of congenital anal atresia and partial sacral agenesis. At a later age she presented with an anterior meningocele, highly suggestive of Currarino’s triad. We discuss the diagnostic and optional treatment aspects of this case, thereby underlining the urgency for early diagnosis.

## Case presentation

**Fig. 1:**
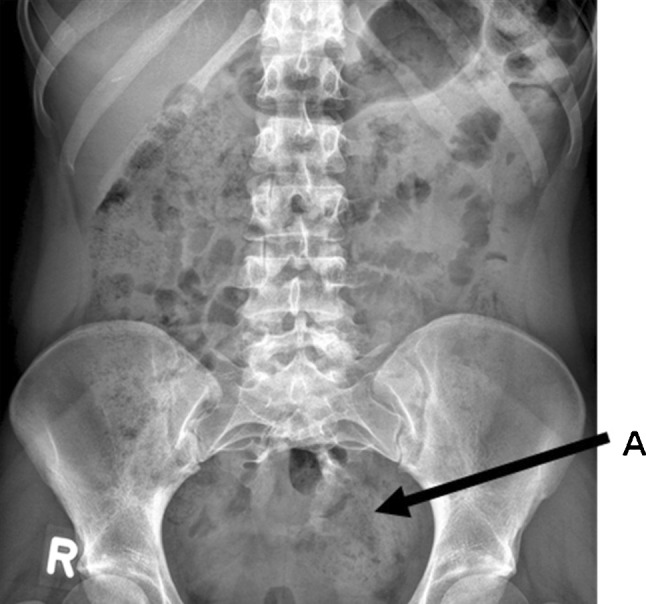
Pelvic X-ray *Arrow A*: the partial sacral agenesis

A 22-year-old Caucasian female was seen in the neurosurgical outpatient clinic. The family history was irrelevant. Her medical history revealed a congenital anal atresia and an ectopic anus. At the age of 3 years, the latter was corrected with an anterior sagittal anorectoplasty (ASARP) procedure. Furthermore, she had suffered from grade III vesico-ureteral reflux. X-ray imaging of the lumbar spine confirmed a partial sacral agenesis, sickle-shaped sacrum (Fig. 1). After surgery, constipation resolved without fecal incontinence. She was followed-up by the pediatric surgeon. Otherwise, she was in healthy condition and is currently employed as a registered nurse.

**Fig. 2:**
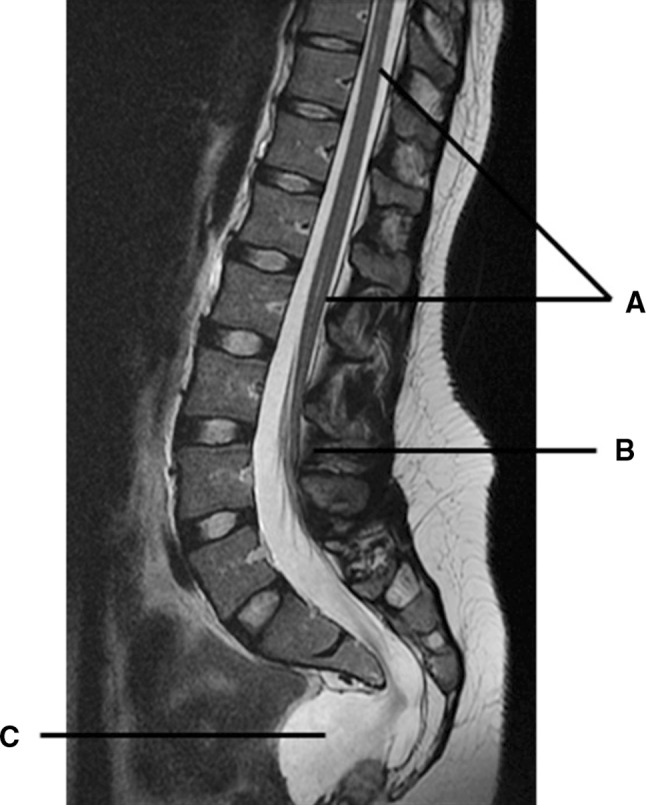
MRI with contrast enhancement. *Arrow A*: mild syringomyelia. *Arrow B*: tethered cord with medullary conus at L3–4 level. *Arrow C*: anterior meningocele

During the second part of 2010, she experienced transient defecation difficulties. For that reason, the pediatric surgical team performed an MRI without contrast of the pelvic region. This showed the formerly known changes as expected from the ASARP procedure and the partial sacral agenesis. Coincidentally an anterior presacral mass of unknown origin, suggestive of a meningocele, as well as a bicornuate uterus was diagnosed (Fig. 2). Neurological examination did not reveal any deficits, explicitly there were neither pyramidal tract nor posterior column signs. The history of anorectal malformations, partial sacral agenesis and the presacral meningocele should prompt to the diagnosis of Currarino’s triad. This syndrome can be accompanied by a tethered cord, and a presacral mass of another origin, such as a teratoma. Therefore, we decided to perform a contrast-enhanced MRI of the lumbosacral spine, to rule out any of the above. It confirmed the presacral mass to be a meningocele. Furthermore, a moderate tethered cord was observed with the medullary conus at level L3–4, as well as a small syrinx. The radiologic findings and the absence of symptoms showed no need for surgical intervention. We decided to pursue a conservative approach and follow her in the outpatient clinic. If complaints or neurological symptoms should occur, depending on the incident pathology, transdural ligation of the meningocele or untethering of the spinal cord could be considered. Genetic counseling was initiated.

## Discussion

Currarino’s triad, although consisting of three groups of abnormalities, includes a wide variability of phenotypes [5]. It is usually diagnosed in childhood and females seem to be more affected [2, 5]. Recognition of the typical features of the triad and the Currarino syndrome itself is important for patient and family counseling. The earlier mentioned homebox gene HLXB9 on locus 7q36 [1, 6] was described various times in sacrococcygeal anomalies including partial sacral agenesis [7]. This mutation can be familial, or incidental [2, 8, 9]. Evaluation of the genetic status and screening family members is strongly advised.

The anorectal malformations occurring in this syndrome often include anal stenoses, sometimes accompanied by Hirschsprung’s disease, or otherwise includes a form of anal agenesis or a ventrally displaced anus [2, 7, 8, 10]. Usually this translates into frequent constipation, as shown in our case, and painful defecation, a common symptom of Currarino’s syndrome [2, 8]. Dilatation can be considered if laxatives or enemas do not solve this condition [2]. Our patient was treated with an ASARP. Literature describes cases mainly treated with a posterior (PSARP) procedure. The reason to choose a posterior approach is that the anorectoplasty is often performed in combination with ligation of the meningocele [5, 11]. For anorectoplasty only, an ASARP procedure leads to comparable results as a PSARP. However, cosmetic satisfaction is higher and a trend of functional benefits has been found [12]. In this case, the anal atresia and ectopic anus present at birth were complicated by several urinary tract infections due to vesicoureteral reflux. Literature reports on other cases with recurrent urinary tract infections, which were usually caused by a neurogenic bladder dysfunction.

The sacrococcygeal agenesis in Currarino’s triad is either complete, in which the sacrum is absent, or partial in which a hemisacrum with preservation of the first sacral vertebra. The latter is often scimitar-shaped and was observed in our patient. A lumbosacral X-ray (Fig. 1) is a good diagnostic tool to evaluate these sacral anomalies [2, 11].

Furthermore, our patient was diagnosed with a presacral mass of unknown origin at a later stage in life. A pelvic MRI was performed because of recurrent constipation in adulthood. Coincidentally a presacral mass was visualized (Fig. 2). MRI of the lumbosacral region is recommended to identify and differentiate the origin of the presacral mass, as well as other associated malformations. It can distinguish a teratoma (type IV) from an anterior meningocele, in view of the fact that the content of the cyst is characteristic of cerebrospinal fluid. A CT myelography can be helpful to illustrate relation to the thecal sac. Treatment of a presacral mass depends on the suspected diagnosis and symptoms. Since teratoma holds malignant potential, depending on maturity, this urges surgical excision with additional chemotherapy. Also in other cases like dermoid cyst, lipoma, leiomyosarcoma, or other malignant masses [5, 7], excision is recommended. Adson et al. described the posterior approach to the resection of an anterior meningocele. Via a lumbosacral partial laminectomy or laminoplasty, a transdural ligation of the neck of the meningocele can be performed [2, 13]. An alternative could be the posterior sagittal approach in between anus and coccyx, which is performed to remove the mass and concomitantly reconstruct the anus [9, 14].

Other common features of Currarino’s syndrome are a tethered cord and syringomyelia, both present in the case presented [2, 15–19]. The incidence of a tethered cord varies highly between presented series from 18 to 83 % [2, 5, 19]. In our patient the conus medullaris was at L3–4 level, but may vary between patients [20]. Possible treatment options are a conservative observation or untethering through a (posterior) laminoplasty approach [21]. Nevertheless, no consensus exists on the surgical treatment of non-symptomatic patients. Some surgeons recommend to perform surgery [2], whereas, others emphasize the risk of surgical complications in an otherwise non-symptomatic patient. More evidently, when progressive pyramidal tract signs or bladder dysfunction is observed, surgery is highly recommended to prevent further worsening.

Besides the described features, Currarino’s syndrome can be accompanied by other pathologies like gynaecological abnormalities [5], reflected by a bicornuate uterus. Also other neurological anomalies may occur, one being holoprosencephaly, a condition in which the prosencephalon fails to develop into two hemispheres and which is related to a defect gene on chromosome 7q36 [22–24]. Therefore, in suspected cases, radiological cerebral examination is recommended.

## Conclusions

Currarino’s triad is a diagnosis made by physical and radiological examination, and diagnosis in adulthood is rare. Young patients with anorectal malformations or other suspicious symptoms should be further investigated. Essential elements include the pelvic X-ray, and lumbar and sacral MRI. Early diagnosis of Currarino’s triad is important to be able to monitor these patients closely and prevent them from developing neurological deficits or worsening conditions. Decisions on treatment depend on two major aspects, namely the occurrence of (neurological) symptoms, and the radiological findings. We recommend an early and multidisciplinary approach if a patient is suspected to have Currarino’s syndrome.
